# Editorial: Rising stars in vascular physiology: 2022

**DOI:** 10.3389/fphys.2023.1278632

**Published:** 2023-09-08

**Authors:** David A. Tulis, Alister J. McNeish

**Affiliations:** ^1^ Department of Physiology, Brody School of Medicine, East Carolina University, Greenville, NC, United States; ^2^ Sosei Heptares, Cambridge, United Kingdom

**Keywords:** cardiovascular disease, inflammation, migration, omega-3 fatty acids, phenotype, proliferation, transcription factor, vascular smooth muscle

Cardiovascular disease (CVD) remains a leading cause of morbidity and mortality worldwide. In order to make inroads in our battle against CVD, we must continually expand our research efforts in pursuit of knowledge regarding all aspects of CVD. Thus, to fully advance our understanding of the many facets of CVD with the hopes of identifying improved treatment options, we need to examine the cardiovascular system under both normal and pathologic conditions. In this light, we were excited to develop a Research Topic entitled *Rising Stars in Vascular Physiology* that aimed to recognize future leaders in vascular physiology and pathology research. This compilation of original research articles from recognized investigators in early stages of their careers offers innovative insights into various aspects of vascular physiology with direct implications to vascular pathology. The emerging areas of interest highlighted by these promising early career scientists include identification and characterization of important transcriptional players in phenotypic modulation and plasticity of vascular smooth muscle (VSM); assessment of the coordination between perivascular nerve physiology and vascular inflammation in neuro-immune signaling during inflammatory bowel disease; identification of novel genes involved in immune-related processes during aortic valve disease and elucidation of their promise as potential clinical biomarkers; and examination of ion channel-specificity for vasodilatory mechanisms of cardioprotective dietary omega-3 fatty acids. These original research findings help shed light on various aspects of vascular physiology and provide potential therapeutic perspectives into vascular pathology.

Normally differentiated, unstimulated VSM cells maintain a quiescent, contractile phenotype, ensuring proper homeostatic functions. However, under stimulated conditions as exist in disease or injury, VSM cells undergo phenotypic modification to a growth-promoting, synthetic state. While phenotypic switching may have benefits under various biological conditions such as tissue re-growth or wound repair, this change plays a fundamental role in the pathogenesis of CVD. Unfortunately, transcriptional programs that help define VSM phenotype including modulation to a growth state remain unclear. In this Research Topic, studies by Alajbegovic et al. and Francisco et al. identified novel capacities of endogenous transcription factors in controlling VSM cell migration and proliferation, characteristics of a growth phenotype, in the context of CVD. In human coronary VSM cells using adenoviral (Ad-) over-expression (OE) of GATA6, a highly conserved zinc finger transcription factor, Alajbegovic et al. identified capacity of GATA6 to promote cell migration. Further, results from microarray analysis suggest that GATA6 operates through transforming growth factor beta (TGF-β)/Smad and mitogen-activated protein kinase signals to promote migration. Paradoxically, these authors also discovered that GATA6 OE maintained cells in a differentiated phenotype through induced expression of the VSM markers MYH11, CNN1, SYNPO2, and ACTA2, thereby providing evidence that differentiation and migration/chemotaxis may not be mutually exclusive. Francisco et al. used Ad- OE of total and phosphorylated SMAD3 and total and phosphorylated FOXO3 in rat primary VSM cells and identified capacities of SMAD3 to induce proliferation and FOXO3 to inhibit proliferation. Interestingly, using cytosolic and nuclear cell fractions and singular or combined SMAD3/FOXO3 OE, results suggest a reciprocal relationship exists between SMAD3 and FOXO3 in their abilities to control VSM growth; SMAD3 reverses the cytostatic effects of FOXO3 and FOXO3 normalizes SMAD3-induced growth. Observations also provided evidence that SMAD3/FOXO3 operate via involvement of the E3 ubiquitin ligase MuRF-1 in VSM cell growth control.

Complementing these studies on phenotypically altered VSM cell migration and proliferation, modified immune responses including enhanced localized inflammation are also central to CVD pathology. Grunz et al. used RNA sequencing and an inflammatory bowel disease (IBD) mouse model to study neuro-immune signaling between adventitial macrophages and perivascular nerves of mesenteric arteries. They found that IBD increased the abundance of adventitial leukocytes and macrophages as well as macrophage-associated genes believed to contribute to impaired vessel dilation; they also observed diminished blood flow via action on sensory nerves. Thus, Grunz et al. provide the first evidence that expansion of the adventitial macrophage population and macrophage-associated gene pool adversely affect perivascular nerve function and contribute to reduced vascular blood flow in IBD. Another study by Lv et al. used data mining in a bioinformatics approach to identify primarily macrophage-associated, immune-related differentially expressed genes (DEGs) in human calcific aortic valve diseased (CAVD) tissues. Algorithmic associations identified key immune-related genes including predominantly SCG2, a chromogranin-secretogranin family member of neuroendocrine secretory proteins, and CCL19, a specific ligand for chemokine receptor CCR7, as candidate biomarkers for CAVD.

Lastly, despite the established role of omega-3 polyunsaturated fatty acids (n-3 PUFAs) in cardiovascular health benefits, there is a lack of clarity regarding key mechanisms in mediating these effects. Bercea et al. used both pharmacological inhibitors and genetic mouse model deficient in VSM K_ATP_ channels to verify whether n-3 PUFA-mediated vasodilation involves opening of these channels. While results from pharmacologic channel blockade suggested that n-3 PUFA-mediated vessel dilation involved K_ATP_, results from knockout animals demonstrated that they were not involved. Thus, the study suggests vascular studies with commonly used pharmacological blockers of K_ATP_ channels need to be interpreted with caution.

Altogether, these timely findings from intriguing and innovative research studies led by young investigators have added to our body of knowledge regarding both vascular physiology and disease processes. As “Rising Stars” in the field, these future leaders, nominated by their peers, have demonstrated ingenuity and insight in their respective studies and should serve as examples for other emerging scientists as well as established investigators in the field of vascular physiology.

